# The impact of migration-related characteristics on the risk of TORCH infections among women of childbearing age: a population-based study in southern China

**DOI:** 10.1186/s12889-023-15238-1

**Published:** 2023-02-16

**Authors:** Rui Li, Lu Han, Wenxue Xiong, Wenjuan Wang, Chaonan Fan, Mingzhen Li, Xiaohua Liu, Li Ling

**Affiliations:** 1 Faculty of Medical Statistic, School of Public Health, Sun Yat-sen University, 510080 Guangzhou, Guangdong China; 2 NHC Key Laboratory of Male Reproduction and Genetics, Guangdong Provincial Reproductive Science Institute (Guangdong Provincial Fertility Hospital), 510600 Guangzhou, China; 3 Clinical research design division, Clinical research center, Sun Yat-sen Memorial Hospital, Sun Yat-sen University, Guangzhou, Guangdong China

**Keywords:** TORCH infections, Migrants, Healthy migrant effect, Women of childbearing age

## Abstract

**Background:**

TORCH infections are the most common prenatal infections causing congenital malformation and infant mortality, especially in developing countries. Migrant women might be vulnerable to TORCH infections, but little is known about the association between migration-related characteristics and TORCH infection risk. This study aimed to investigate the impact of migrant status, migration distance, and the spouse’s migrant status on the TORCH epidemic among women of childbearing age.

**Methods:**

Based on the National Free Preconception Health Examination Project, we analyzed a representative dataset of TORCH infections among women of childbearing age (15–49 years old) in Guangdong Province of China (2014–2019, *n* = 2,451,297). The past and/or recent infection status of TORCH infections (Toxoplasma gondii [TOX], Cytomegalovirus [CMV], and Rubella virus [RV]) were identified. Demographic and migration-related characteristics were collected. We thoroughly assessed the prevalence of TORCH infections in both migrant and native women and estimated adjusted odd ratios (aOR) for migration-related characteristics using multivariable logistic regression after adjusting the other sociodemographic factors.

**Results:**

Among all 2,451,297 participants, 443,725 (18.1%) were migrant women. Migrant women presented a lower risk of past TOX infection (aOR: 0.89, 0.88–0.91) suggesting a healthy migrant effect (HME), but a higher risk of recent TOX infection (aOR: 1.88, 1.77–1.99), past CMV infection (aOR: 1.26, 1.25–1.28) and RV infection in natural ways (aOR: 1.05, 1.04–1.06). Compared with intra-provincial migrants, inter-provincial migrants had a lower past TOX infection (aOR: 0.88, 0.85–0.91), but a higher risk of recent TOX infection (aOR: 1.16, 1.05–1.27) and RV infection (aOR: 1.33, 1.31–1.36). In addition, having a migrant spouse was associated with a higher risk for all types of infection.

**Conclusion:**

This study reported the association of migrant status and migration distance with TORCH infections, although the significance and directionality of these associations varied between pathogens. The spouse’s migrant status further amplified the infection risk for all types of pathogens. Our findings suggested interventions for preventing the spread of CMV and RV infection and new acquisition of TOX infection for migrants in southern China, to narrow the native-migrant health inequity and decrease the incidence of prenatal infections and related adverse outcomes.

**Supplementary Information:**

The online version contains supplementary material available at 10.1186/s12889-023-15238-1.

## Introduction

TORCH infections refer to perinatal infections caused by serial organisms including Toxoplasma gondii (TOX), Rubella virus (RV), Cytomegalovirus (CMV), Herpes simplex virus (HSV) and others, which are the leading cause of prenatal and infant morbidity and mortality [1, 2]. The epidemiological and clinical features, such as the risks of acquiring an infection, symptoms and sequelae, vary by pathogen and are reviewed for example in [3]. Generally, migrant women are substantially more vulnerable to TORCH infections due to disadvantaged socio-economic conditions [4, 5], low public awareness of prevention measures [6], and lifestyle-related risk behaviors [7]. With the longstanding household registration system in China, migrant women (who remain official resident registration of their communities of origin but moved to other places for at least six months) have typically suffered access barriers to reproductive health care services because of entitlement restrictions linked with local household registration [8]. Guangdong province, located in southern China, has attracted the largest migrant population (52 million migrants in 2020, accounting for 41.3% of the whole population), hence identifying the migrant-native disparities in the prevalence of TORCH infections are urgently needed.

Extensive researches have pointed out the migrants’ economic, physical and sexual vulnerabilities and the increased risk of infectious diseases [9, 10]. However, emerging literature has offered contradictory findings that migrants were in better health than populations in the host country, known as the “Healthy Migrant Effect” (HME) [11, 12]. Ojeda’s analysis reported the protective effect of migrant status for sexually transmitted infections (STIs) acquisition in Mexico [13]. A prior study conducted in China also found migrants at no higher risk of acquiring syphilis or human immunodeficiency virus (HIV) than local dwellers [14]. However, in terms of TORCH infections, the comparison between native and migrant women of childbearing age is insufficient, further studies are needed to evaluate whether the phenomenon of HME also existed. Furthermore, far too little attention has been paid to the heterogeneity of the migrant groups. Migration with different characteristics may further modify the infection risk of TORCH for migrant women. Firstly, for the considerable geographic and cultural disparities across provinces in China, inter-provincial migrants experience more acculturative stress compared to intra-provincial migrants [15]. But the impact of migration distance (the proxy for inter-provincial and intra-provincial migration) on TORCH infections is often overlooked. Secondly, the risks associated with the mobility of the spouse indirectly affected a women’s health status. It has been observed that women had a higher risk of HIV infection [16, 17] and the symptoms of STIs [18] when their spouses had a history of migration. However, as for migrant women, the impact of the spouse’s migrant status on the infection risk has not been precisely and quantitatively assessed.

To fill the aforementioned gaps, the aim of this study was to assess the prevalence of TORCH infections among migrant and native women and furthermore to explore the impact of women’s migrant status, migration distance and the spouse’s migrant status on past and recent infection risk, using a population-based survey in southern China with over 2.4 million women of childbearing age (15–49 years old). Gaining insight into the epidemiology profile of TORCH infections among women of childbearing age and its association with migration-related characteristics are key for developing more extensive and tailored intervention programs and allocating prevention and treatment resources more effectively.

## Methods

### Data source

The National Free Preconception Health Examination Project (NFPHEP), a series of population-based, nationwide cross-sectional surveys, was piloted in 220 rural counties located in 31 provinces and municipalities during 2010–2012 and promoted to both rural and urban areas in the whole country since 2013 [19]. This project has provided 19 preconception health service items involving health education, physical check-up, risk assessment and preconception counseling for married couples who planning to conceive within the coming six months and the coverage rate of the target population exceeded 80%. Serological screenings of TOX, CMV, and RV for women are included in this project to perform early diagnosis and treatment of TORCH infections. More details about the design, organization, implementation, and quality control of this project have been previously described at length [20, 21].

### Research site and study population

Guangdong Province, located on the southeastern coast, is one of the most developed provinces in China and a key destination for migrants. The East Asian monsoon is the main climatic type in this area, where the light, heat, and water resources are rich. This study was based on the rounds of NFPHEP conducted from January 2014 to September 2019 in Guangdong Province. 2,679,011 women aged from 15 to 49 years were included in this study, then those who did not have serological testing for TOX, CMV, and RV before pregnancy, those with missing data on the migrant status, and duplicated recodes were excluded. Overall, 2,451,297 participants (91.5%) were included in the final analysis (**Fig S1**).

### Identification of classification of migration-related characteristics

Three migration-related characteristics were included in this research: migrant status, migration distance, and the spouse’s migrant status. Based on the Chinese household registration system, migrants refer to individuals who move from the place where they were born to other areas of the country without possessing the local “hukou” (residence registration certificates) for more than six months [22]. To be included in the NFPHEP survey, all participants were required to have stayed in the locale for at least 6 months. In this study, migrant women therefore were identified by matching the current resident county with their household registered county, and the migrant status of their spouse was defined similarly. In other words, two types of migrant status were recognized: natives (those with local hukou) and migrants (those living in the locale for more than 6 months and without local hukou registration). Considering the similarity of the cultural and geographic environment within the province, we grouped migrant women according to their migrant distance: Intra-provincial and Inter-provincial migrants. Specifically, migrants who resided in the county and with the place of household registration in Guangdong Province were classified as “Intra-provincial migrants”. Conversely, migrants with the place of household registration in other provinces except Guangdong were classified as “Inter-provincial migrants”. In addition, migrants were also categorized by the migrant status of their spouse into “Migrants with native spouse” and “Migrants with migrant spouse”. Specifically, the former referred to migrant women whose spouses have local “hukou” at the current residence and the latter referred to migrant women whose spouses were also identified as migrants.

### Covariables

**Fig. 1:**
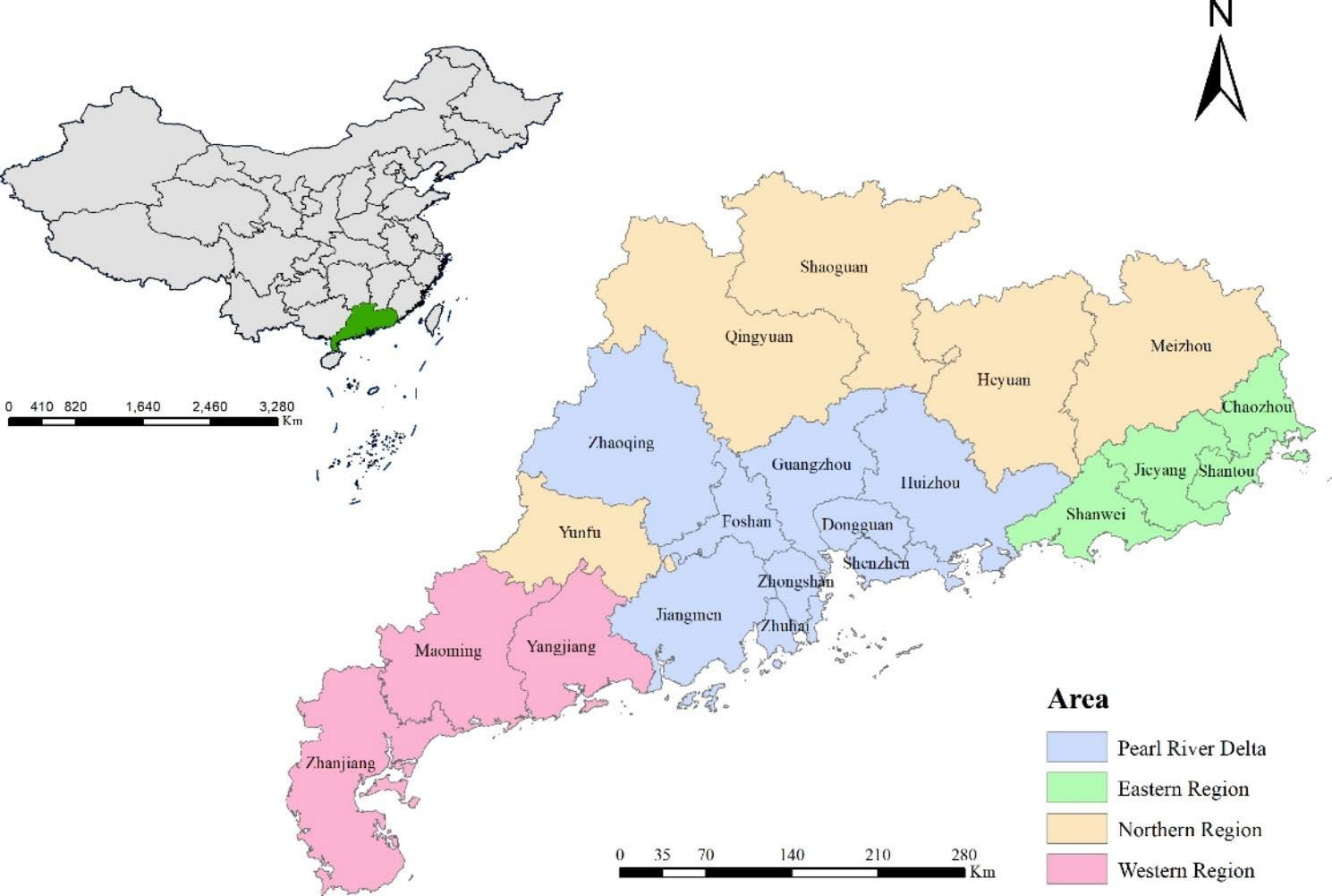
**Location of Guangdong province in China and the economic geographical division of Guangdong.** Base layers of the maps were downloaded from Resource and Environment Science and Data Center (http://www.resdc.cn/data.aspx?DATAID=201).

Referring to previous surveys [23–25], Covariates were selected to control for potential confounding related to sociodemographic characteristics, including age, ethnicity, educational attainment, occupation, and residential region. The age of participants was grouped into 15–19, 20–24, 25–29, 30–34, 35–39, 40–44, and 45–49 years old. The ethnicity was grouped into Han and the minority. The education level was divided into primary school or below, junior high school, senior high school, and college or higher. The occupation was classified into workers, farmers, homemakers, businesswomen, those working in the service industry, civil servants, and others. The unemployed and job-waiting women were subsumed into the category of “others”. Twenty-one municipal cities in Guangdong were divided into four residential regions based on geographical location and economic characteristics: Northern region (Heyuan, Meizhou, Qingyuan, Shaoguan, and Yunfu), Eastern region (Shanwei, Shantou, Chaozhou, and Jieyang), Western region (Zhanjiang, Yangjiang, and Maoming) and Pearl River Delta region (Guangzhou, Shenzhen, Zhuhai, Foshan, Huizhou, Dongguan, Zhongshan, Jiangmen and Zhaoqing) (Fig. 1). Dummy variables for each study year were also included, considering possible changes in the social and economic environment over the study period.

### Outcomes

Serum specimens with concentrations of anti-TOX IgG > 8.8 IU/mL and anti-TOX IgM > 10 AU/mL, anti-CMV IgG > 22.0 IU/mL, anti-CMV IgM > 18 AU/mL and anti-RV IgG > 10.0 IU/mL, were considered as positive. Typically, IgM antibodies develop within approximately 1–2 weeks after the onset of the infection and their levels rise until peaking after 1–3 months and then gradually decline to undetectable levels [26]. In contrast, the IgG antibodies develop more slowly and persist for life thereafter [27]. Referring to the previous study [23, 28], IgG+/IgM- indicated the past infection beyond at least 6 months of a specific pathogen for the participants, and IgM+/(IgG- or IgG+) indicated the recent infection in this study [29].

### Statistical analyses

Sociodemographic characteristics were presented as numbers and percentages (%) and significant differences between native and migrant women were assessed by the Chi-square test. We obtained estimates of the prevalence with 95% confidence intervals (CI) for recent and/or past infections of TOX, CMV, and RV. Logistic regression was performed to calculate the adjusted odds ratio (aOR) with 95% CI of each group of migrants with respect to the native women group (reference group), for all types of infections studied. To explore the internal heterogeneity of the migrant groups, the aOR with 95% CI of inter-provincial migrants (reference group: intra-provincial migrants) and migrant women with migrant spouses (reference group: migrant women with native spouses) for past and recent TOX, CMV and RV infections were also calculated. We further conducted the stratified analysis by age group and residential region. All tests were two-sided, and *P* values less than 0.05 were considered statistically significant. The statistical analysis was conducted using R version 4.0.3.

## Results

### Descriptive statistics

Among 2,451,297 participants who were finally included in the analyses, 2,007,572 (81.9%) were native women and 443,725 (18.1%) were migrant women. The median age of all participants was 27 years (Interquartile range: 24–30). Table 1 shows the sociodemographic characteristics of the entire study sample for native women and migrant women. Groups differed with regard to variables including age, ethnicity, educational level, occupation, residential region, and the spouse’s migrant status (all *P* values < 0.001). In both groups, most participants were between 25 and 29 years old, of Han ethnicity, and had an education level of college or above. Although the majority of participants in both groups were living in the Pearl River Delta region, the percentage of native women living there was only 44.4% while for migrant women it was 75.0%. Among migrant women, 51.6% of them were intra-provincial migrants. As for the spouse’s migrant status, 35.4% of migrant women had a migrant spouse while only 10.3% of native women had a migrant spouse. Participants excluded from the current analysis owing to missing information had similar sociodemographic characteristics (**Table S1**).

**Table 1:** Sociodemographic characteristics of native and migrant women of childbearing age in Guangdong, 2014–2019

	Native women			Migrant women		*P*
	*n* ^a^	%		*n* ^a^	%	
**All**	2,007,572	81.9		443,725	18.1	
**Age**						< 0.001
15–19	584	0.0 ^b^		72	0.0 ^b^	
20–24	569,532	28.4		101,778	22.9	
25–29	874,337	43.6		216,112	48.7	
30–34	340,028	16.9		90,207	20.3	
35–39	151,187	7.5		28,026	6.3	
40–45	56,769	2.8		6341	1.4	
45–49	15,135	0.8		1189	0.3	
**Ethnicity**						< 0.001
Han	1,915,805	99.5		423,513	97.0	
Minority	9613	0.5		12,923	3.0	
**Educational level**						< 0.001
Primary school or below	45,734	2.6		6748	1.6	
Junior high school	592,766	33.8		108,445	26.4	
Senior high school	471,442	26.9		112,928	27.4	
College or above	643,022	36.7		183,291	44.6	
**Occupation**						< 0.001
Workers	429,001	25.5		126,127	31.7	
Farmers	463,518	27.5		49,002	12.3	
Homemakers	68,312	4.1		14,427	3.6	
Businesswomen	85,301	5.1		23,511	5.9	
Service industry	170,161	10.1		49,409	12.4	
Civil servants	376,804	22.4		113,264	28.4	
Others	91,753	5.4		22,444	5.6	
**Residential region**						< 0.001
Northern	338,023	16.8		38,239	8.6	
Eastern	473,820	23.6		51,118	11.5	
Western	303,571	15.1		21,578	4.9	
Pearl River Delta	892,158	44.4		332,790	75.0	
**Migration distance**						-
Intra-provincial	2,007,572	100.0		229,001	51.6	
Inter-provincial	-	-		214,724	48.4	
**Spouse’s migrant status**						
Native	1,799,825	89.7		286,590	64.6	< 0.001
Migrant	206,267	10.3		156,855	35.4	

^a^ Missing exists if the sum of n is less than N

^b^ The proportion is less than 0.05 and has been rounded

### Prevalence of TORCH infections and its association with migrant status

**Fig. 2:**
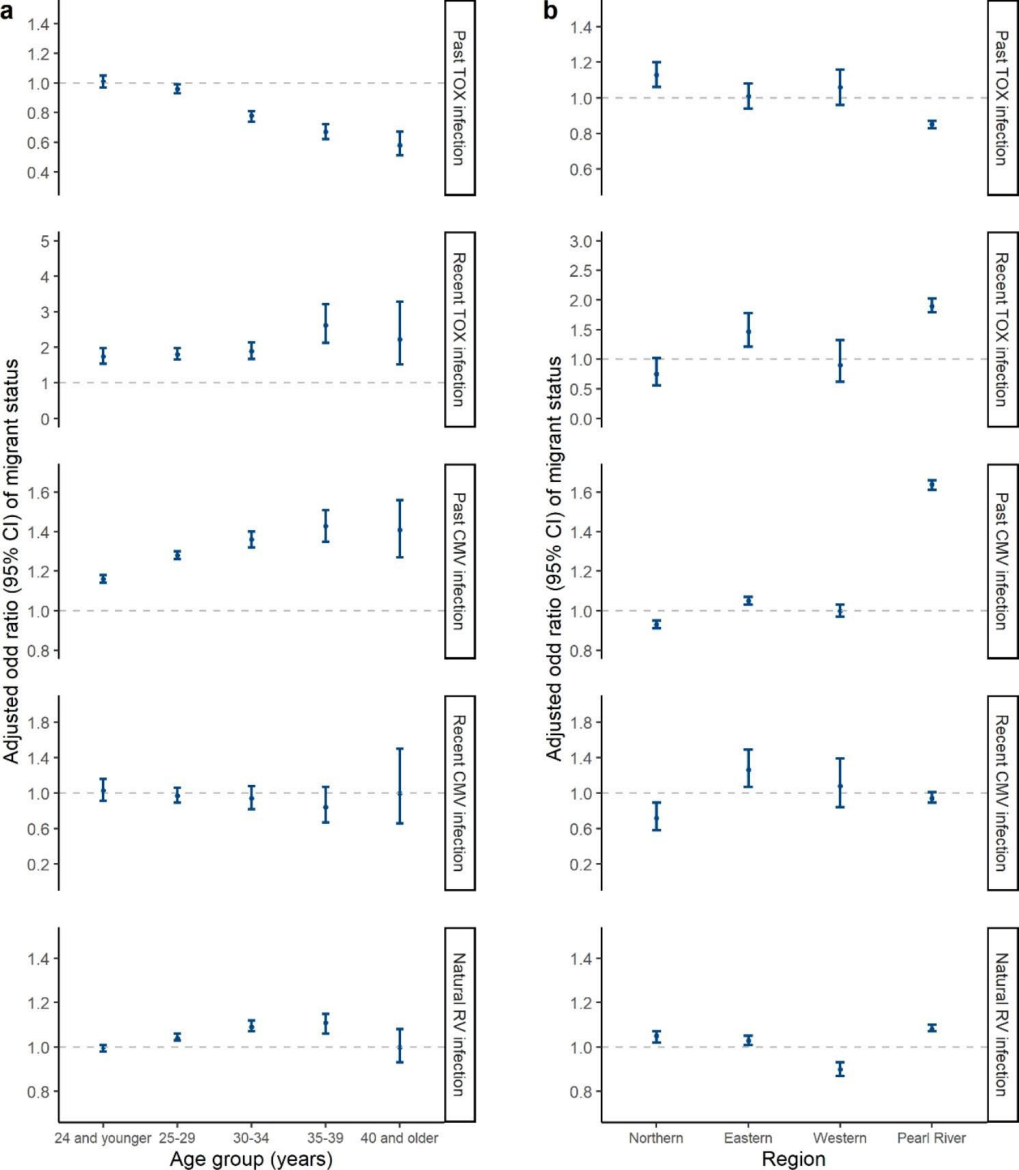
**Adjusted odd ratios of migrant status stratified by age group and residential region.** (a) The figure shows multivariable-adjusted odds ratios for the prevalence among migrant women compared with that among native women, stratified by age groups. Ethnicity, educational attainment, occupation, residential address, and study year were included as covariates. 15–19 and 45–49 age groups were merged into adjacent age groups to increase the statistical power. (b) The figure shows multivariable-adjusted odds ratios for the prevalence among migrant women compared with that among native women, stratified by residential regions. Age group, ethnicity, educational attainment, occupation, and study year were included as covariates

The overall prevalence of TOX, CMV and RV IgG + among the 2,451,297 women of childbearing age were 3.20% (95% CI: 3.18–3.23%), 77.52% (95% CI: 77.47–77.57%) and 75.90% (95% CI: 75.86–75.95%), respectively. The positive rate of TOX and CMV IgM + were 0.27% (95% CI: 0.26–0.27%) and 0.35% (95% CI: 0.35–0.36%), respectively. The prevalence of past and recent TOX and CMV infections and the differences between native and migrant women are shown in Table 2. For TOX, the prevalence rate of past infection was 3.39% (95% CI: 3.34–3.44%) among migrant women and 3.10% (95% CI: 3.07–3.12%) among native women (aOR: 0.89, 95% CI: 0.88–0.91). Anti-TOX IgM antibody was positive in 2114 (0.48%, 95% CI: 0.46–0.50%) migrants and 4383 (0.22%, 95% CI: 0.21–0.22%) natives (aOR: 1.88, 95% CI: 1.77–1.99). It indicated that migrant women were less likely to be previously infected with TOX, but more likely to be recently infected. For CMV, migrant women had higher odds of past infection with CMV compared with native women (aOR: 1.26, 95% CI: 1.25–1.28), while the odds of recent infection among migrants and natives showed no significant difference (aOR: 0.96, 95% CI: 0.91–1.02). Table 3 shows that the self-reported RV vaccination rate among native women (6.10%, 95% CI: 6.07–6.13%) was significantly higher than that among migrant women (4.85%, 95% CI: 4.78–4.97%) (χ^2^ = 1032.1, *P* < 0.001). Of the total reporting no vaccination against RV or unsure about their history, anti-RV IgG antibody was positive in 335,509/422,224 (79.46%, 95% CI: 79.35–79.57%) migrant women and 1,420,162/1,885,139 (75.33%, 95% CI: 75.28–75.39%) native women (aOR: 1.05, 95% CI: 1.04–1.06), suggesting that migrants were more likely acquired immunity by natural infection. Notably, inter-provincial migration and the spouse’s migrant status further exaggerated the native-migrant prevalence gaps mentioned above. Figure 2 shows the multivariable-adjusted odds ratios for migrant status stratified by age groups and residential regions. Similar associations between migrant status and the infection risk of the above pathogens were found in almost all age groups (Fig. 2a) and the Pearl River Delta region (Fig. 2b).

**Table 2:** Prevalence of TOX and CMV infections and their association with migrant status

	No. of Positive/No. of Total	Prevalence (95% CI)	Adjusted OR ^a,b^ (95% CI)
**Past infection**			
**Anti-TOX IgG+/IgM-**			
All migrants	15,042/443,725	3.39 (3.34–3.44)	0.89 (0.88–0.91)
Intra-provincial	7954/229,001	3.47 (3.40–3.55)	0.96 (0.94–0.99)
Inter-provincial	7088/214,724	3.30 (3.23–3.38)	0.82 (0.80–0.85)
Native spouse	8863/286,590	3.09 (3.03–3.16)	0.91 (0.89–0.94)
Migrant spouse	6166/156,855	3.93 (3.84–4.03)	0.86 (0.84–0.89)
Natives	62,201/2,007,572	3.10 (3.07–3.12)	Reference
**Anti-CMV IgG+/IgM-**			
All migrants	379,277/443,725	85.48 (85.38–85.57)	1.26 (1.25–1.28)
Intra-provincial	191,238/229,001	83.51 (83.36–83.66)	1.21 (1.19–1.23)
Inter-provincial	188,039/214,724	87.57 (87.43–87.71)	1.37 (1.35–1.39)
Native spouse	230,556/286,590	80.45 (80.32–80.58)	1.09 (1.08–1.10)
Migrant spouse	148,455/156,855	94.64 (94.53–94.76)	2.49 (2.43–2.56)
Natives	1,514,434/2,007,572	75.44 (75.38–75.49)	Reference
**Recent infection**			
**Anti-TOX IgM+/(IgG- or IgG+)**			
All migrants	2114/443,725	0.48 (0.46–0.50)	1.88 (1.77–1.99)
Intra-provincial	849/229,001	0.37 (0.35–0.40)	1.57 (1.45–1.70)
Inter-provincial	1265/214,724	0.59 (0.56–0.62)	2.23 (2.08–2.39)
Native spouse	832/286,590	0.29 (0.27–0.31)	1.25 (1.16–1.36)
Migrant spouse	1279/156,855	0.82 (0.77–0.86)	2.99 (2.77–3.22)
Natives	4383/2,007,572	0.22 (0.21–0.22)	Reference
**Anti-CMV IgM+/(IgG- or IgG+)**			
All migrants	1687/443,725	0.38 (0.36–0.40)	0.96 (0.91–1.02)
Intra-provincial	870/229,001	0.38 (0.36–0.41)	0.99 (0.92–1.07)
Inter-provincial	817/214,724	0.38 (0.35–0.41)	0.93 (0.86–1.01)
Native spouse	1051/286,590	0.37 (0.34–0.39)	0.99 (0.92–1.07)
Migrant spouse	636/156,855	0.41 (0.37–0.44)	0.91 (0.82–0.99)
Natives	7008/2,007,572	0.35 (0.34–0.36)	Reference

Abbreviation: *aOR* Adjusted odds ratio, *CI* confidence interval, *TOX* Toxoplasma gondii, *CMV* Cytomegalovirus, *IgG* Immunoglobulin G, *IgM* Immunoglobulin M

^a^ Separate models of all migrant women compared to native women; inter-provincial migrant women and intra-provincial migrant women compared to native women; migrant women with migrant spouses and migrant women with native spouses compared to native women were constructed

^b^ Adjusted ORs were calculated by multivariate logistic regression after adjusting for age, ethnicity, educational attainment, occupation, residential address, and study year

**Table 3:** Prevalence of RV vaccination and seroconversion and their association with migrant status

	No. of Positive/No. of Total	Prevalence (95% CI)	Adjusted OR ^a,b^ (95% CI)
**Self-reported vaccine history**			
All migrants	21,501/443,725	4.85 (4.78–4.91)	0.78 (0.77–0.80)
Intra-provincial	11,373/229,001	4.97 (4.88–5.05)	0.80 (0.79–0.82)
Inter-provincial	10,128/214,724	4.72 (4.63–4.81)	0.76 (0.75–0.78)
Native spouse	15,633/286,590	5.45 (5.37–5.54)	0.89 (0.87–0.90)
Migrant spouse	5854/156,855	3.73 (3.64–3.83)	0.60 (0.58–0.61)
Natives	122,433/2,007,572	6.10 (6.07–6.13)	Reference
**Anti-RV IgG + by natural infection** ^**c**^			
All migrants	335,509/422,224	79.46 (79.35–79.57)	1.05 (1.04–1.06)
Intra-provincial	166,197/217,628	76.37 (76.19–76.55)	0.92 (0.91–0.93)
Inter-provincial	169,312/204,596	82.75 (82.59–82.92)	1.24 (1.22–1.25)
Native spouse	208,631/270,957	77.00 (76.86–77.14)	1.00 (0.99–1.01)
Migrant spouse	126,659/151,001	83.88 (83.69–84.06)	1.15 (1.13–1.17)
Natives	1,420,162/1,885,139	75.33 (75.28–75.39)	Reference

Abbreviation: *aOR* Adjusted odds ratio, *CI* confidence interval, *RV* Rubella virus, *IgG* Immunoglobulin G

^a^ Separate models of all migrant women compared to native women; inter-provincial migrant women and intra-provincial migrant women compared to native women; migrant women with migrant spouses and migrant women with native spouses compared to native women were constructed

^b^ Adjusted ORs were calculated by multivariate logistic regression after adjusting for age, ethnicity, educational attainment, occupation, residential address, and study year

^c^ Anti-RV IgG + by natural infection was conducted among women who reported not having RV vaccination

**The impact of migration distance and the spouse’s migrant status on the infection risk among migrants**.

In multivariable logistic regressions that controlled for other covariates (Table 4), we found that migration distance had significant associations with the prevalence of TOX and RV infection among migrant women (all *P* < 0.001). Inter-provincial migrants were less likely to have a past infection (aOR: 0.88, 95% CI: 0.85–0.91) but more likely to have a recent infection for TOX (aOR: 1.16, 95% CI: 1.05–1.27). Meanwhile, migrant women having a migrant spouse, compared with those having a native spouse, had a significant association with seropositivity of past and recent TOX infections (aOR: 1.12, 95% CI: 1.07–1.16; aOR: 2.24, 95% CI: 2.01–2.50, respectively), past CMV infection (aOR: 2.07, 95% CI: 2.00-2.13), and natural RV infection (aOR: 1.12, 95% CI: 1.09–1.14). Similar results were found in the subgroups of different age groups and residential regions (**Fig S2**). Age, ethnicity, educational attainment, occupation, and residential regions were all associated with the prevalence of TORCH infections but the relationship was not consistently significant for different types of pathogens (**Table S2**).

**Table 4:** The impact of migration distance and the spouse’s migrant status on TORCH infections among migrant women

Variables	Anti-TOX IgG+/IgM-			Anti-TOX IgM+/(IgG- or IgG+)			Anti-CMV IgG+/IgM-			Anti-CMV IgM+/(IgG- or IgG+)			Anti-RV IgG+ by natural infection	
	Adjusted OR	*P*		Adjusted OR	*P*		Adjusted OR	*P*		Adjusted OR	*P*		Adjusted OR	*P*
**Migration distance**														
Intra-provincial	1	< 0.001		1	< 0.001		1	0.67		1	0.79		1	< 0.001
Inter-provincial	0.88 (0.85–0.91)			1.16 (1.05–1.27)			1.00 (0.98–1.03)			0.99 (0.88–1.10)			1.33 (1.31–1.36)	
**Spouse’s migrant status**														
Native spouse	1	< 0.001		1	< 0.001		1	< 0.001		1	0.38		1	< 0.001
Migrant spouse	1.12 (1.07–1.16)			2.24 (2.01–2.50)			2.07 (2.00-2.13)			0.95 (0.83–1.07)			1.12 (1.09–1.14)	

Abbreviation: *aOR* Adjusted odds ratio, *CI* confidence interval, *TOX* Toxoplasma gondii, *CMV* Cytomegalovirus, *RV* Rubella virus, *IgG* Immunoglobulin G, *IgM* Immunoglobulin M

Notes:

1) aORs were calculated by multivariate logistic regression after adjusting for age, ethnicity, educational attainment, occupation, residential address, and study year

2) IgG+/IgM- indicated that the participant had been previously infected with pathogens, IgM+/(IgG- or IgG+) indicated that the participants had recently been infected with the pathogens

3) Anti-RV IgG + by natural infection was conducted among women who reported not having RV vaccination

4) The reference groups of aOR were intra-provincial migrant women and migrant women with native spouses, respectively

## Discussion

To our knowledge, this is the first study to thoroughly examine the impact of migration-related characteristics on the prevalence of CMV, TOX, and RV infections among women of childbearing age with an unprecedentedly large dataset of over 2.4 million samples in southern China. Overall, compared with native women, migrants had a higher prevalence of recent TOX infection, past CMV infection, and RV infection by natural ways, but a lower risk of past TOX infection suggesting a healthy migrant effect. We also found that inter-provincial migrants had a lower risk of past TOX infection but a higher risk of recent TOX infection and past RV infection than intra-provincial migrants. Having a migrant spouse for migrant women further amplifies the risk of TOX, CMV, and RV infections. This study helps to formulate tailored intervention programs for preventing prenatal infections and improving maternal and infant health in this resource-constrained setting.

### Migrant status and TORCH infections

Surprisingly, migrant women showed a lower risk of past TOX infection as compared with native women, which could be explained by several mechanisms connected to the healthy migrant effect (HME). The seroprevalence of past infection among the migrant population generally reflects the prevalence of TOX infection in their places of origin, because migration is a recent fact [29]. Previous studies observed significant regional variations in the prevalence of human TOX infection with the trends increasing from West China to East China, which coincided with the incidence of TOX infection in food animals [24, 30]. As an economically developed province, Guangdong has attracted a huge number of migrant populations from the less developed central and western regions. Migration connected areas of low and high risk, leading to the lower prevalence of past TOX infection among migrants. Meanwhile, the fact that people with better health status are led to migrate might also explain this protective effect. However, a higher infection risk of recent TOX infection among migrants than natives was found in this study. This implied that the healthy migrant effect might be offset as individuals acculturate to local customs in the new host place. The adoption of negative lifestyle factors such as eating raw seafood in Guangdong might increase potential exposure to infectious agents [31, 32]. These findings implied that migrants were at high risk of TOX acquisition after coming into Guangdong. Thus, reducing the transmission from local sources of infection to migrants might play a critical role in the prevention of primary TOX infection during preconception.

Unlike TOX infection, there was little support for the existence of an HME when it comes to CMV and RV. We identified a higher rate of past CMV infection among migrant women, which was consistent with several previous findings [7, 33]. This might have been attributed to the transmission of infection through physical contact and increased sexual risk behaviors during the migration process [34, 35]. In addition, this study found that migrant women had a lower vaccination rate of RV and were more prone to have a past infection of RV by natural ways. Previous studies have reported the insufficient utilization of health care services among migrants than general populations due to restrictive health-related policies, poor economic conditions, and the lack of awareness of seeking medical services [36, 37]. Migrants were often overlooked for the RV vaccine catch-up immunization programs and their immunity was often acquired by natural infection [38]. Therefore, immunization strategies targeting migrants were urgently needed, which proved beneficial in preventing the spread of infection and guaranteeing migrant health in the European countries [39].

### Migration distance and TORCH infections among migrants

In terms of the impact of migration distance on the infection risk of TOX, this study discovered that inter-provincial migrants had a lower risk of past infection but a higher risk of recent infection than intra-provincial migrants. This finding, similar to the results about the impact of migrant status, further implied that the burden of TOX infection may be predominantly attributed to local parasite prevalence, dietary habits, and cultural habits, rather than the importation of latent infections acquired by migrants from other provinces. The abundant natural water network and ample annual precipitation in Guangdong possibly help the oocyst spread and retain accessible for potential hosts [30], posing threat to susceptible women like inter-provincial migrants. Moreover, inter-provincial migrants were more prone to be infected with RV in natural ways than intra-provincial migrants. The literature on the immunization status of RV among rural Chinese women pointed out the relatively high RV vaccination rate in Guangdong than elsewhere [40]. A reduction in the force of infection due to vaccination partly restrained the acquisition of RV infection among intra-provincial migrants.

### The spouse’s migrant status and TORCH infections among migrants

Insight on how the spouse’ migrant status influences TORCH infections among migrant women have not been studied as extensively. One previous study on maternal CMV serostatus in early pregnancy suggested that both maternal and paternal migrant status have been reported to be correlated with the presence of CMV-specific IgG antibodies in the maternal serum and there was also an interaction between them [41]. Similarly, this study indicated that migrant women had a greater risk of TOX, CMV, and RV infections when their spouses also were migrants. Reports of the presence of TOX, CMV, and RV in semen, saliva, and cervical secretions and several lines of epidemiological evidence have suggested that sexual activity facilitates the transmission of the above pathogens [42–44]. Besides, women whose spouses were migrants were significantly at higher risk of being infected with sexually transmitted diseases [17, 18, 45]. Thus, it is reasonable that among migrant women, the infection of TOX, CMV, and RV may also be affected by their spouse’s migrant status, which suggested that interventions targeted at the spouse of childbearing aged women are also an essential part of managing TORCH infections.

### Strengths and limitations

Relying on the NFPHEP, this study has reliable data, a large sample size, and good sample representation, allowing us to perform a convincing comparison between different populations. Moreover, this study thoroughly assessed the native-migrant gaps in the prevalence of TOX, CMV, and RV infections and emphasized the role of migrant distance and the spouse’s migrant status in the disease transmission. From the broader public health perspective, this study helps to develop effective responses to improve maternal and infant health. Identifying vulnerable women of childbearing age can guide the implementation of targeted screening strategies and prophylaxis measures.

There are several limitations to this study. Firstly, the serological results of IgG and IgM antibodies cannot indicate the precise timing of infection, and the order of migration and past and recent infection cannot be defined. As the presence of IgM antibodies indicates recent infections and one of the inclusion criteria of NFPHEP was staying in the locale for at least 6 months, it’s reasonable to speculate that recent infections happened in the hosting place after migration. The seroprevalence of past infection among the migrant population generally reflects the prevalence of the infection in their places of origin[29]. But for individuals with long migration duration, the past infection may happen at the hosting place, which might lead to the underestimation of the migrant-native disparity in past infection risk. Secondly, sociodemographic information and vaccination history of RV were self-reported and may be subject to measurement error. Thirdly, a cross-sectional design for this study cannot establish causality between migration-related characteristics and TORCH infections. But standardized laboratory TORCH testing enables us to identify more precisely the prevalence of infection among native and migrant women. Fourthly, the household registration transfer records were unavailable, and migrant women who converted to local “hukou” were misclassified as native women, which may lead to the underestimation of the difference between native and migrant women. Finally, other TORCH pathogens, such as HSV and syphilis were not included in this study.

## Conclusion

In the present study, we detected a higher risk of recent TOX infection, past CMV infection, and RV infection in migrant women compared to native women. Conversely, the lower risk of past TOX infection in migrants, especially inter-provincial migrants, was also observed, suggesting a healthy migrant effect. Moreover, the spouse’s migrant status further amplified the infection risk in migrant women for all types of pathogens. The findings of this study suggest that narrowing the health gap by intervention programs targeted to migrants (especially for inter-provincial migrants and those with the migrant spouse), such as preconception screening, catch-up immunization and health education programs, would reduce CMV and RV infection. More attention to controlling local sources of TOX infection in Guangdong and reducing the transmission to susceptible migrant women is urgently needed. Understanding potential mechanisms linking migration and TORCH infections is meaningful for reducing the risk of congenital infection and promoting maternal and infant health in China and other counties facing migration-related issues.

## Electronic supplementary material

Below is the link to the electronic supplementary material.

Supplementary Material 1

## Data Availability

Data are available upon reasonable request via email to the author (sysuhanlu@126.com).

## List of abbreviations

TOX: Toxoplasma gondii
CMV: Cytomegalovirus
RV: Rubella Virus
HSV: Herpes Simplex Virus
IgG: Immunoglobulin G
IgM: Immunoglobulin M
HME: Healthy Migrant Effect
NFPHEP: National Free Preconception Health Examination Project
CI: Confidence Interval
OR: Odds Ratio
