# Supplementary material for: The impact of migration-related characteristics on the risk of TORCH infections among women of childbearing age: a population-based study in southern China
Source: BMC Public Health. 2023 Feb 16;23:351. doi: 10.1186/s12889-023-15238-1 (PMC9933307; doi:10.1186/s12889-023-15238-1)
Supplement: Supplementary file 1 — Supplementary Material 1 [file 12889_2023_15238_MOESM1_ESM.docx]

**Supplementary Material**

**The impact of migration-related characteristics on the risk of TORCH infections among women of childbearing age: A population-based study in southern China**

Rui Li, Lu Han, Wenxue Xiong, Wenjuan Wang, Chaonan Fan, Mingzhen Li, Xiaohua Liu, Li Ling

**Fig S1.** Flowchart of Participant Selection

**Fig S2.** The associations between migration distance, the spouse’s migrant status, and the risk of TORCH infections stratified by age group and residential region.

**Table S1.** Sociodemographic characteristics of participants included or excluded from analysis due to missing information

**Table S2.** Covariables from multivariable logistic regression. Each column represents adjusted OR and corresponding CI displayed for each variable for each type of antibody

This supplementary material has been provided by the authors to give readers additional information about their work.


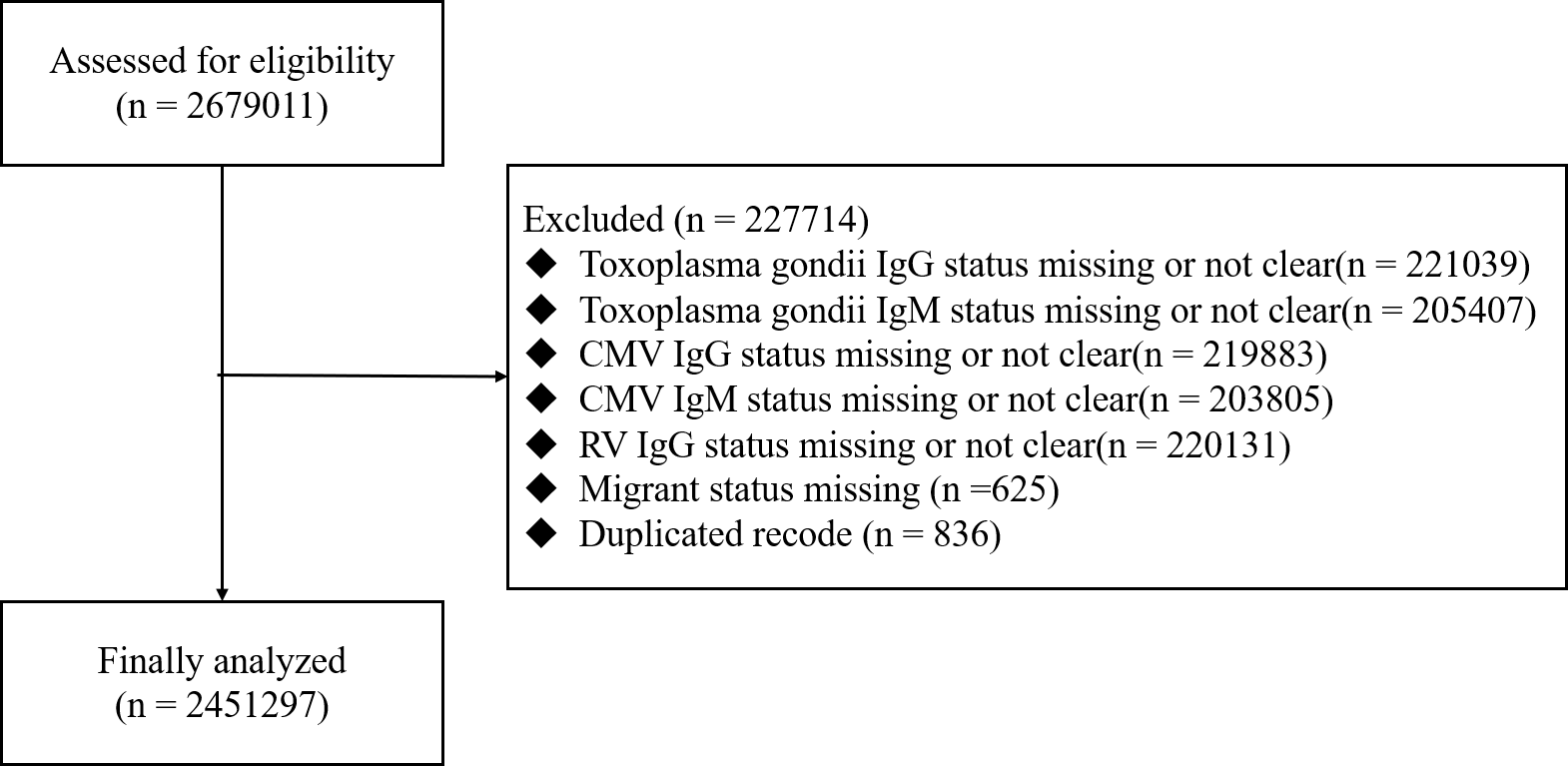


**Fig S1.** **Flowchart of Participant Selection**


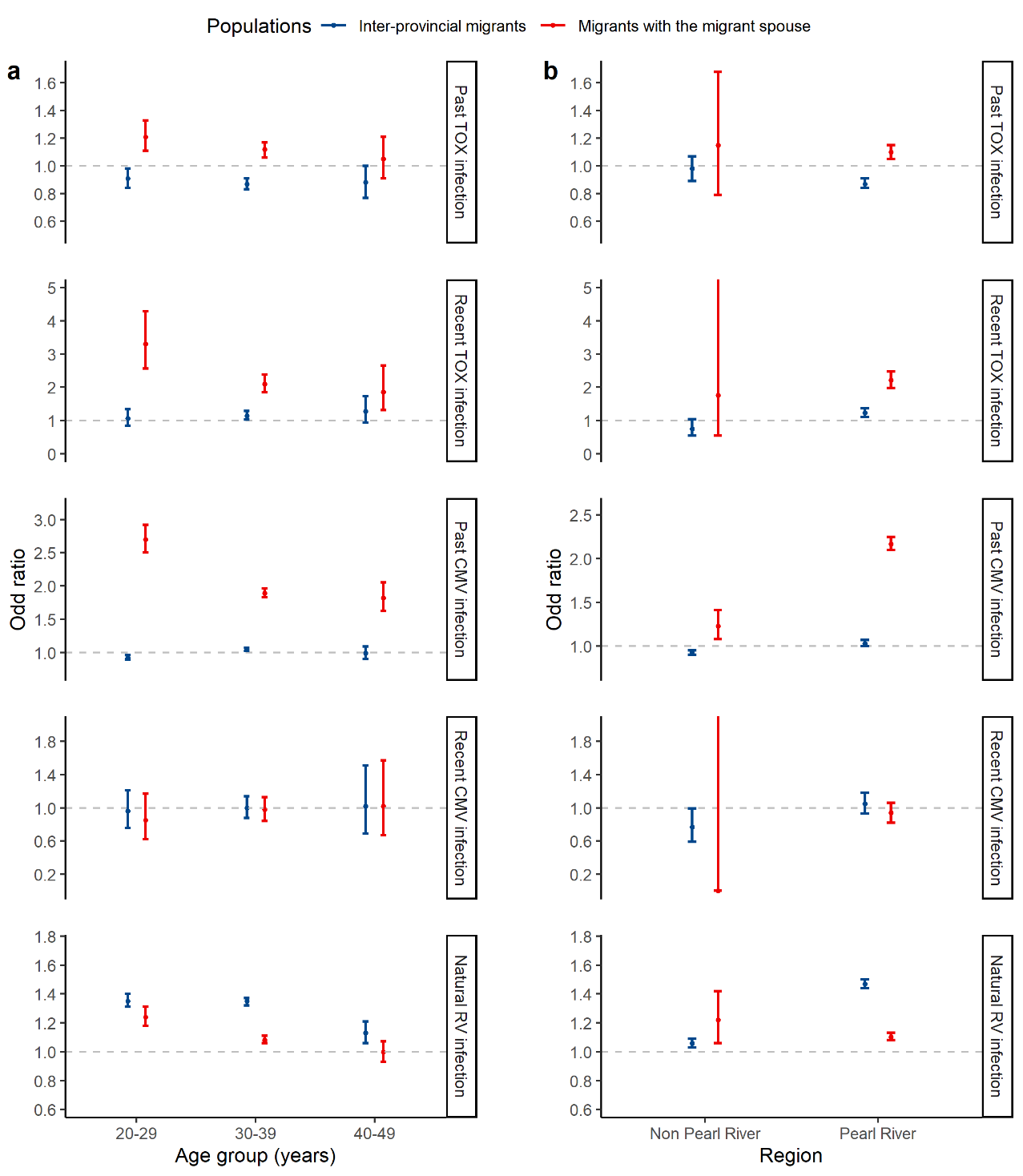


**Fig S2.** **The associations between migration distance, the spouse’s migrant status, and the risk of TORCH infections stratified by age group and residential region.** Intra-provincial migrants and having a native spouse were the reference group respectively. **a** The figure shows multivariable-adjusted odds ratios of inter-provincial migrants and having a migrant spouse stratified by age groups. **b** The figure shows multivariable-adjusted odds ratios of inter-provincial migrants and having a migrant spouse stratified by residential region. Part of the upper limit of 95% CI was not shown because of out of bound.

**Table S1.** **Sociodemographic characteristics of participants included or excluded from analysis due to missing information**

|  | Included participants | |  | Excluded participants | |
| --- | --- | --- | --- | --- | --- |
|  | *n* ^a^ | % |  | *n* ^a^ | % |
| **All** | 2451297 |  |  | 227011 |  |
| **Age** |  |  |  |  |  |
| 15-19 | 656 | 0.0 ^b^ |  | 147 | 0.1 |
| 20-24 | 671310 | 27.4 |  | 68261 | 30.1 |
| 25-29 | 1090449 | 44.5 |  | 94102 | 41.5 |
| 30-34 | 430235 | 17.6 |  | 39868 | 17.6 |
| 35-39 | 179213 | 7.3 |  | 16520 | 7.3 |
| 40-45 | 63110 | 2.6 |  | 6320 | 2.8 |
| 45-49 | 16324 | 0.7 |  | 1793 | 0.8 |
| **Ethnicity** |  |  |  |  |  |
| Han | 2339318 | 99.0 |  | 187965 | 99.2 |
| Minority | 22536 | 1.0 |  | 1510 | 0.8 |
| **Educational level** |  |  |  |  |  |
| Primary school or below | 52482 | 2.4 |  | 3520 | 2.4 |
| Junior high school | 701211 | 32.4 |  | 56457 | 38.4 |
| Senior high school | 584370 | 27.0 |  | 38278 | 26.0 |
| College or above | 826313 | 38.2 |  | 48782 | 33.2 |
| **Occupation** |  |  |  |  |  |
| Workers | 555128 | 26.6 |  | 29662 | 21.6 |
| Farmers | 512520 | 24.6 |  | 46392 | 33.7 |
| Homemakers | 82739 | 4.0 |  | 6579 | 4.8 |
| Businesswomen | 108812 | 5.2 |  | 6163 | 4.5 |
| Service industry | 219570 | 10.5 |  | 12924 | 9.4 |
| Civil servants | 490068 | 23.5 |  | 28473 | 20.7 |
| Others | 114197 | 5.5 |  | 7363 | 5.4 |
| **Residential region** |  |  |  |  |  |
| Northern | 376262 | 15.3 |  | 38791 | 17.1 |
| Eastern | 524938 | 21.4 |  | 40675 | 17.9 |
| Western | 325149 | 13.3 |  | 41557 | 18.3 |
| Pearl River Delta | 1224948 | 50.0 |  | 105988 | 46.7 |
| **Migration distance** |  |  |  |  |  |
| Intra-provincial | 229001 | 51.6 |  | 18603 | 54.6 |
| Inter-provincial | 214724 | 48.4 |  | 15452 | 45.4 |
| **Spouse’s migrant status** |  |  |  |  |  |
| Native | 2086415 | 85.2 |  | 197844 | 87.2 |
| Migrant | 363122 | 14.8 |  | 28930 | 12.8 |

^a^ Missing exists if the sum of n is less than N.

^b^ The proportion is less than 0.05 and has been rounded.

**Table S2. Covariables from multivariable logistic regression. Each column represents adjusted OR and corresponding CI displayed for each variable for each type of antibody**

| Variables | Anti-TOX  IgG+/IgM- | Anti-TOX  IgM+/(IgG- or IgG+) | Anti-CMV  IgG+/IgM- | Anti-CMV  IgM+/(IgG- or IgG+) | Anti-RV IgG+  by natural infection |
| --- | --- | --- | --- | --- | --- |
| **Age group** |  |  |  |  |  |
| 15-19 | Ref | Ref | Ref | Ref | Ref |
| 20-24 | 1.24  (0.30-5.09) | 0.09***  (0.03-0.31) | 0.72  (0.36-1.43) | 0.23  (0.03-1.70) | 0.99  (0.55-1.77) |
| 25-29 | 1.07  (0.26-4.38) | 0.10***  (0.03-0.32) | 0.80  (0.40-1.59) | 0.24  (0.03-1.72) | 1.24  (0.69-2.22) |
| 30-34 | 1.03  (0.25-4.20) | 0.10***  (0.03-0.33) | 0.91  (0.46-1.82) | 0.24  (0.03-1.73) | 1.33  (0.75-2.38) |
| 35-39 | 1.04  (0.25-4.28) | 0.11***  (0.03-0.35) | 1.06  (0.53-2.13) | 0.23  (0.03-1.65) | 1.29  (0.72-2.31) |
| 40-45 | 1.04  (0.25-4.31) | 0.09***  (0.03-0.31) | 1.02  (0.51-2.05) | 0.26  (0.03-1.96) | 1.23  (0.68-2.20) |
| 45-49 | 0.85  (0.20-3.63) | 0.08***  (0.02-0.34) | 0.94  (0.45-1.97) | 0.25  (0.03-2.30) | 1.33  (0.73-2.44) |
| **Ethnicity** |  |  |  |  |  |
| Han | Ref | Ref | Ref | Ref | Ref |
| Minority | 1.17***  (1.06-1.29) | 1.38***  (1.12-1.70) | 0.92***  (0.87-0.98) | 0.93  (0.68-1.27) | 0.94*  (0.90-0.99) |
| **Educational level** |  |  |  |  |  |
| Primary school or below | Ref | Ref | Ref | Ref | Ref |
| Junior high school | 0.90  (0.79-1.03) | 0.87  (0.62-1.23) | 0.95  (0.89-1.01) | 1.18  (0.77-1.80) | 1.03  (0.97-1.09) |
| Senior high school | 0.81***  (0.71-0.93) | 0.86  (0.61-1.21) | 1.12***  (1.05-1.20) | 1.08  (0.70-1.64) | 1.18***  (1.11-1.25) |
| College or above | 0.71***  (0.62-0.81) | 0.69*  (0.49-0.97) | 1.18***  (1.11-1.26) | 1.04  (0.68-1.60) | 1.20***  (1.13-1.28) |
| **Occupation** |  |  |  |  |  |
| Workers | Ref | Ref | Ref | Ref | Ref |
| Farmers | 1.04  (0.98-1.10) | 1.07  (0.92-1.25) | 1.04*  (1.01-1.07) | 0.92  (0.77-1.09) | 1.01  (0.99-1.04) |
| Homemakers | 0.95  (0.87-1.05) | 1.29*  (1.05-1.59) | 1.22***  (1.16-1.29) | 0.84  (0.63-1.11) | 1.04  (1.00-1.09) |
| Businesswomen | 0.89***  (0.82-0.96) | 1.18  (0.98-1.41) | 1.17***  (1.12-1.23) | 0.95  (0.76-1.18) | 0.97  (0.93-1.00) |
| Service industry | 0.96  (0.91-1.02) | 0.81**  (0.69-0.95) | 1.20***  (1.16-1.24) | 0.73***  (0.61-0.87) | 1.15***  (1.11-1.18) |
| Civil servants | 0.91***  (0.86-0.95) | 0.78***  (0.68-0.89) | 1.44***  (1.40-1.49) | 0.69***  (0.59-0.80) | 1.24***  (1.21-1.27) |
| Others | 0.95  (0.88-1.03) | 1.01  (0.82-1.23) | 1.29***  (1.23-1.35) | 1.05  (0.85-1.30) | 1.08***  (1.04-1.12) |
| **Residential region** |  |  |  |  |  |
| Northern | Ref | Ref | Ref | Ref | Ref |
| Eastern | 0.61***  (0.56-0.67) | 2.14***  (1.5-3.05) | 0.99  (0.96-1.02) | 1.49***  (1.14-1.96) | 1.05***  (1.02-1.09) |
| Western | 0.80***  (0.71-0.89) | 1.08  (0.65-1.79) | 1.19***  (1.15-1.24) | 1.42*  (1.01-1.99) | 0.99  (0.95-1.03) |
| Pearl River Delta | 1.41***  (1.32-1.51) | 3.50***  (2.54-4.82) | 4.77***  (4.63-4.91) | 1.96***  (1.54-2.50) | 1.61***  (1.57-1.66) |

Abbreviation: *OR* Odds ratio, *CI* Confidence interval, *TOX* Toxoplasma gondii, *CMV* Cytomegalovirus, *RV* Rubella virus, *IgG* Immunoglobulin G, *IgM* Immunoglobulin M.

Notes:

1) Adjusted ORs of the study year were not shown. *** represents *P* < 0.001; ** represents *P* < 0.01; * represents *P* < 0.05.

2) IgG+/IgM- indicated that the participant had been previously infected with pathogens, IgM+/(IgG- or IgG+) indicated that the participants had recently been infected with the pathogens.

3) Anti-RV IgG+ by natural infection was conducted among women who reported not having RV vaccination.
